# Longitudinal Changes in Posttraumatic Stress Disorder After Resettlement Among Yazidi Female Refugees Exposed to Violence

**DOI:** 10.1001/jamanetworkopen.2021.11120

**Published:** 2021-05-28

**Authors:** Jana Katharina Denkinger, Caroline Rometsch, Martha Engelhardt, Petra Windthorst, Johanna Graf, Phuong Pham, Niamh Gibbons, Stephan Zipfel, Florian Junne

**Affiliations:** 1 Department of Psychosomatic Medicine and Psychotherapy, University Hospital Tübingen, Tübingen, Germany; 2 Alice Salomon University Berlin, University of Applied Science, Berlin, Germany; 3 Department of Psychosomatic Medicine and Psychotherapy, Hospital Havelhöhe, Berlin, Germany; 4 Harvard Humanitarian Initiative, Harvard University, Cambridge, Massachusetts; 5 Department of Psychosomatic Medicine and Psychotherapy, University Hospital Magdeburg, Otto von Guericke University, Magdeburg, Germany

## Abstract

**Importance:**

Posttraumatic stress disorder (PTSD) is highly prevalent among refugees surviving mass atrocities, especially among women. Longitudinal studies investigating factors associated with PTSD course are essential to enable adequate treatment yet widely lacking.

**Objective:**

To identify longitudinal changes in PTSD severity and posttraumatic coping among severely traumatized female refugees as well as risk and protective factors for PTSD course.

**Design, Setting, and Participants:**

This prospective cohort study took place in 14 German cities in the context of a humanitarian admission program that resettled 1000 especially vulnerable women and children from northern Iraq to Germany. Approximately 400 adult beneficiaries of the humanitarian admission program were eligible for the study. At baseline, a total of 116 of the 400 beneficiaries (29.0%) participated, with 96 (82.8%) of these women participating in the follow-up assessment. The study included a baseline assessment conducted 2 years after resettlement (September 1, 2017, to January 12, 2018) and a 1-year follow-up (August 29, 2018, to January 15, 2019).

**Exposures:**

Violence and/or captivity during the 2014 genocide in northern Iraq by the so-called Islamic State.

**Main Outcomes and Measures:**

Posttraumatic stress disorder severity and coping strategies were assessed in interpreter-aided interviews using the Impact of Event Scale–Revised.

**Results:**

A total of 116 women (mean [SD] age, 32.2 [8.2] years; 115 [99.1%] Yazidi; 1 [0.9%] Christian) participated at baseline. According to the Impact of Event Scale–Revised, a high PTSD severity was found (mean [SD] raw sum score, 60.88 [15.75] of 88, with higher scores indicating greater distress), with no significant change over time. Helpful coping strategies included prayer, belief in collective strength, and belief in personal strength. Earlier symptoms of intrusions (β = 0.389, *P* = .007) and longer captivity (β = 0.218, *P* = .02) were identified as being associated with PTSD severity 1 year later. Longer captivity was associated with PTSD aggravation over time (β = 0.227, *P* = .04). Posttraumatic strengthening in faith (β = −0.206, *P* = .05) and in social relationships (β = −0.221, *P* = .03) were associated with a reduction in PTSD symptoms.

**Conclusions and Relevance:**

These findings suggest that female refugee survivors of genocide are at high risk for severe and chronic PTSD beyond the initial years of resettlement. The findings provide suggestions for mental health care specialized for particularly vulnerable populations.

## Key Points

**Question:**

How does posttraumatic stress disorder (PTSD) change during a 1-year period in female refugees who survived mass atrocities, and what factors are associated with PTSD course?

**Findings:**

This cohort study with 116 female refugee survivors of captivity and genocide found high PTSD severity 2 years after resettlement in Germany with no significant change 1 year later. Factors associated with severe PTSD were earlier symptoms of intrusions and longer time spent in traumatic situations, whereas strengthening in faith and social relationships were associated with symptom relief over time.

**Meaning:**

This study suggests that female refugee survivors of genocide and captivity are at high risk for severe and chronic PTSD beyond the initial years of resettlement.

## Teaser

This cohort study assesses longitudinal changes in posttraumatic stress disorder severity and posttraumatic coping among severely traumatized Yazidi female refugees, as well as risk and protective factors for posttraumatic stress disorder course.

## Introduction

Mental illnesses, such as posttraumatic stress disorder (PTSD), are prevalent after mass atrocities and displacement. Early studies^1,2,3,4,5,6,7^ investigating 1 of the most recently affected populations, displaced Yazidis from northern Iraq after the 2014 genocide, indicate an alarming prevalence of PTSD (42.9%-100%), with women having higher prevalence rates than men. Previous research^8,9,10,11,12^ with genocide survivors (eg, in Rwanda or Bosnia) found that severe mental health effects can last years, even decades, after genocide.

The greater prevalence of PTSD among women is also seen in genocide-affected and refugee populations.^10,13,14^ A potential explanation might be that sexualized violence is disproportionately committed against women in armed conflicts.^15,16,17^ This explanation aligns with the recently published finding that exposure to gender-based violence, including sexual slavery while held in captivity by the nonstate armed organization known as the Islamic State (IS), was associated with PTSD in Yazidi women after the 2014 genocide.^18^

Although several factors associated with PTSD onset have been identified, studies investigating factors associated with the longitudinal PTSD course, meaning the trajectory of posttraumatic symptoms over several years, remain rare.^19^ A systematic review^19^ of naturalistic prospective cohort studies with trauma survivors found social relationships and support to be preventive of a severe, chronic PTSD symptom trajectory. The review^19^ also found that female sex, older age, minority status, trauma severity, and PTSD symptoms (particularly higher hyperarousal) at baseline are associated with an unfavorable PTSD course. Other studies^20,21,22,23,24^ have yielded inconsistent findings regarding whether individual PTSD symptoms are associated with subsequent PTSD. Because only a few studies in the systematic review^19^ involved genocide survivors or refugees and other cross-sectional studies^25,26^ indicate the existence of distinct PTSD symptomatologic patterns in refugees, a deeper understanding of the course of PTSD and potential risk and resilience factors is essential for this field of research and clinical practice.

Previous cross-sectional studies^27,28^ have highlighted the effective use of coping strategies (eg, sense of purpose in life, use of social support, or religious coping) as preventive for PTSD after trauma and as associated with lower PTSD symptoms and even PTSD recovery. Coping refers to cognitive and behavioral efforts to help an individual master, reduce, or tolerate specific external and/or internal demands that are perceived as overwhelming.^29,30^ Two early qualitative studies^31,32^ with small samples suggest collective and religious coping strategies to be salient among Yazidi genocide survivors.

Current understanding of the longitudinal course of PTSD and the effectiveness of coping strategies is incomplete, particularly with regard to the distinctive needs of female refugees after surviving mass atrocities. Longitudinal studies in this area are essential for research and clinical practice. Because such studies are currently lacking, the current study combines 2 main aims. The first aim, following a pathogenic approach, is to identify the course of PTSD in resettled female survivors of the 2014 genocide and potential factors associated with PTSD severity and course over time. The second aim, following a salutogenic approach, is to identify preferred coping strategies and protective factors during the long-term course of PTSD in this high-risk group. On the basis of the previous research discussed above,^19,27,28,31,32^ we hypothesized that earlier PTSD symptoms and higher severity of trauma exposure (for Yazidi women, the amount of time spent in IS captivity) are associated with an unfavorable PTSD course. Positive posttraumatic changes in religious and social factors are hypothesized to be significantly associated with a favorable PTSD course.

## Methods

### Study Design

The prospective cohort study included a baseline (September 1, 2017, to January 12, 2018) and a 1-year follow-up (August 29, 2018, to January 15, 2019) assessment. The study proceeded via interpreter-aided interviews. Results of the baseline investigation regarding psychosomatic symptoms and perspectives on justice are presented in previous publications.^33,34^ The Harvard Human Research Protection Program Institutional Review Board and the clinical Ethics Committee of the University Hospital Tübingen approved the study. Participants gave written informed consent to participate and to be contacted again 1 year later for a follow-up assessment. All data were deidentified. This study followed the Strengthening the Reporting of Observational Studies in Epidemiology (STROBE) reporting guideline.^35^

### Participants and Setting

In August 2014, IS attacked and overran the Sinjar Mountains of the Nineveh governorate in northern Iraq. The mountain region has historically been home to the Yazidis (Êzidî), who were explicitly and brutally targeted by IS.^36^ This extreme violence included enslavement, forced conversion to Islam, rape, forced marriage, and execution^2,37^ and was declared to be an ongoing genocide in 2016 by the United Nations.^38^ In response, the German federal state Baden-Württemberg implemented a humanitarian admission program (HAP) called Special Quota Project for Especially Vulnerable Women and Children From Northern Iraq, which was open to women and children without family support who had survived IS violence, especially sexual assault.^39,40^ Before resettlement, approximately 98% of the HAP beneficiaries met the criteria for PTSD.^41,42^ From March 17, 2015, through January 2016, a total of 1000 women and children were flown out of Iraq to Germany.^40^ The beneficiaries received secure housing in 19 cities in Baden-Württemberg and financial support. Moreover, medical, psychotherapeutic, and psychiatric support was available to the women.^39,43,44,45^ In our sample, approximately 73% had seen a psychologist in the first 2 years of resettlement.^39^

All adult HAP beneficiaries were eligible to participate in the current study because they were already preselected as severely affected by trauma through the program. Social workers of each HAP accommodation center were invited to inform their clients about participation starting from July 2017. Voluntary recruitment within this special sample aimed to enable participation without creating pressure. When women confirmed interest, the research team provided detailed study information in Kurdish-Kurmanji, Arabic, or German. All 116 study participants had experienced the 2014 IS attacks in northern Iraq.

### Survey Instruments

#### Impact of Event Scale–Revised

To measure PTSD severity, the Impact of Event Scale–Revised (IES-R)^46,47^ was used. This self-report measure consists of 22 items that assess the degree of distress caused by PTSD symptoms during the past 7 days on a 5-point Likert scale (0 to 4), with 0 indicating not at all and 4 indicating extremely. The IES-R raw sum scores (ranging from 0 to 88) as well as 3 subscale scores (intrusions, avoidance, and hyperarousal) can be derived, with higher scores indicating higher levels of distress. All 3 subscale scores range from 0 to 4, with the intrusion subscale score consisting of the mean of 8 item scores (eg, regarding intrusive thoughts or nightmares), the avoidance subscale score referring to the mean of another 8 item scores (eg, regarding the avoidance of feelings or reminders), and the hyperarousal subscale score defined as the mean of 6 item scores (eg, regarding irritability).^47^ The IES-R shows high internal consistency (α = .96).^48^

Even though the IES-R is not a diagnostic tool, there is evidence that it might discriminate between individuals with and without probable PTSD, and cutoff scores have been cited in previous literature.^49^ Best diagnostic accuracy was found with a cutoff score of 33.^48^ Because this cutoff has been used in previous literature within different samples, including refugees,^48,50,51^ we report the percentage of IES-R raw sum scores above 33 to enable comparison with other studies.

#### Context-Specific Questionnaire Items and Coping

To assess context-specific details, the study team, consisting of epidemiologists, psychologists, psychotherapists, and physicians from Harvard University and the University Hospital of Tübingen experienced in the research field of genocide, developed questionnaire items that contained sociodemographic characteristics (eg, ethnicity), HAP specifics (eg, time spent in captivity), and potential outcomes associated with trauma (eg, changes in faith through trauma and feelings of exclusion from the community) (eAppendix in the Supplement). Moreover, the perceived helpfulness of different emotion-focused coping strategies according to Folkman and Lazarus^29,30^ in the aftermath of trauma was assessed (“How much do the following strategies help you cope with the effects of IS violence? 1. Belief in collective strength, e.g. strength of the Yazidi community or your family, 2. Belief in personal strength, e.g., belief in yourself and your own strength, 3. Praying, 4. Social retreat, e.g., spending time alone, 5. Exchange trauma contents with others, 6. Seeking professional help, e.g., doctors, psychotherapist, 7. Seeking help within the Yazidi community”). Questionnaire items were answered on a 5-point Likert-scale (0 to 4), with 0 indicating not at all and 4 indicating extreme, or as open-ended questions (ie, when asking about ethnicity or religion). During follow-up, the same questionnaire was used with some additions (eg, 1 item assessing changes in social relationships during the past year).

The questionnaire was developed in German and English and then translated into Kurdish-Kurmanji, the language spoken by participants. The Kurdish translations were discussed, revised, and agreed on by a multidisciplinary expert team that included Kurdish and Yazidi members. The final version was piloted and discussed with 2 Kurdish/Yazidi women to ensure comprehensibility and cultural appropriateness.

#### Study Implementation

Interviewers were female mental health professionals accompanied by female interpreters. Before data collection, interviewers and interpreters received several days of training. Interviews took place in private rooms of the HAP accommodation in 14 German cities. To facilitate the selection of responses to quantitative questions and to ensure accuracy, participants could show their answer on a graphic representation of the Likert scale. At baseline, interviews were audio-recorded, and the Kurdish segments of the recordings, instead of the interpreters’ spontaneous translations, were translated again and transcribed to validate the data entry process and to allow the research team to analyze the interviews qualitatively.^39^

### Statistical Analysis

For sample description, means, valid percentages, and distributions are reported. Repeated-measure analyses of variance were calculated with Greenhouse-Geisser adjustments for lack of sphericity and Bonferroni-adjusted post hoc analyses to counteract the problem of multiple comparisons. Furthermore, Pearson correlations, independent *t* tests, Mann-Whitney tests, and paired-samples *t* tests were used. A first multiple linear regression analysis was performed to test the hypothesis that the severity of distinct PTSD symptom clusters at baseline is associated with overall PTSD severity 1 year later. Because previous studies^20,21,22,23,24^ suggest that past PTSD severity is associated with subsequent PTSD severity but findings can be inconsistent regarding individual PTSD symptom clusters as factors associated with subsequent PTSD, we included all 3 baseline IES-R subscale scores as independent variables, controlling for age^52,53^ and number of days spent in captivity.^42^ Given that the authors of the IES-R recommend the use of means of the different item scores rather than raw sum scores^46^ and that we aimed to avoid an overrepresentation of the 8-item subscales over the 6-item subscale in the sum score for PTSD severity for this analysis, we used the sum of all 3 subscales (IES-R subscale sum score range, 0-12)^54,55^ at follow-up instead of the IES-R raw sum score as the dependent variable. A second multiple linear regression was performed to test the hypothesis that posttraumatic changes in faith and in social relationships are associated with a favorable PTSD course,^19^ even when age^52,53^ and the number of days in captivity are controlled for.^42^ The PTSD course over time was defined as the difference of IES-R subscale sum scores at follow-up and baseline (change in IES-R subscale sum scores = IES-R subscale sum score at follow-up − IES-R subscale sum score at baseline). Multicollinearity was assessed with the values of tolerance and the variance of inflation factor. All tests were 2-sided with a significance level of α = .05. Statistical analyses were performed using SPSS statistical software, version 24 (IBM Inc).

## Results

### Demographic Information

At baseline, 116 women (mean [SD] age, 32.2 [8.2] years; 115 [99.1%] Yazidi; 1 [0.9%] Christian) participated in the study. With approximately 400 adult HAP beneficiaries, the estimated response rate of this study was 29% at minimum. At baseline, participants had been in Germany for approximately 2 years. One year later, 96 individuals (82.8%) participated in the follow-up assessment. Four participants dropped out because they moved to other cities in Germany and 3 others because they were in Iraq at the time of data collection. Other reasons for dropout were limited time resources and health issues. Sociodemographic characteristics of the sample are presented in Table 1.

**Table 1.** Sociodemographic and Trauma-Related Characteristics of the Study Participants^a^

Sociodemographic characteristic	Baseline (n = 116)	Follow-up (n = 96)
Marital status		
Married or partnership	68 (58.6)	62 (64.5)
Single	32 (27.6)	18 (18.8)
Widowed	15 (12.9)	15 (15.6)
Divorced	1 (0.9)	1 (1.0)
Residence of spouse if married		
In Germany with respondent	11 (16.2)	11 (17.7)
In Germany without respondent	6 (8.8)	8 (12.9)
In Iraq	24 (35.3)	18 (29.0)
Other country	3 (4.4)	0
Missing or unknown	24 (35.3)	25 (40.3)
Highest educational level		
None	33 (28.4)	27 (28.1)
Incomplete primary	16 (13.8)	19 (19.8)
Finished primary	50 (43.1)	39 (40.6)
Intermediate	13 (11.2)	10 (10.4)
High school	4 (3.4)	1 (1.0)
Education and profession		
Literacy	73 (62.9)	68 (70.8)
Current school enrollment	96 (82.8)	60 (62.5)
Currently employed	2 (1.7)	7 (7.4)
Seeking employment	23 (19.8)	24 (27.0)
Time since arrival in Germany, mean (SD) [range], d	727.90 (90.07) [539-934]	1091.13 (81.00) [911-1283]
Time spent in captivity, mean (SD) [range], d	202.68 (127.10) [0-720]	NA
IES-R scores, mean (SD) [range]		
IES-R raw sum score	60.88 (15.75) [14-87]	59.98 (14.55) [24-87]
IES-R subscale sum score	8.28 (2.16) [1.83-11.88]	8.16 (2.03) [3-11.88]
Intrusion subscale score^b^	2.93 (0.93) [0.25-4]	2.92 (0.96) [0.25-4]
Hyperarousal subscale score^b^	2.70 (0.91) [0-4]	2.64 (1.03) [0-4]
Avoidance subscale score^b^	2.66 (0.86) [0.13-4]	2.61 (0.89) [0.25-4]
Change in faith attributable to the traumatic event, mean (SD) [range]^c^	2.96 (1.21) [0-4]	2.63 (1.19) [0-4]
Change in social relationships in the past year, mean (SD) [range]^d^	NA	2.57 (1.29) [0-4]

Abbreviations: IES-R, Impact of Event Scale–Revised; NA, not applicable.

^a^ Data are presented as number (percentage) of participants unless otherwise indicated.

^b^ The IES-R subscale scores are associated with symptom severity because the score structure represents the items’ Likert scale (with 0 indicating not at all and 4 indicating extremely).^47^

^c^ Items were rated on a 5-point Likert scale (0-4), with 0 indicating weakened and 4 indicating strengthened.

^d^ Items were rated on a 5-point Likert scale (0-4), with 0 indicating worse and 4 indicating better.

### Initial Assessment

#### Posttraumatic Stress Disorder

Participants had been in captivity for a mean (SD) of 6.8 (4.2) months. At baseline, 2 years after resettlement, a mean (SD) IES-R raw sum score of 60.88 (15.75) was found, with 101 (92.7%) scoring above a cutoff of 33, indicating levels of symptoms associated with probable PTSD in previous studies.^48^ Statistically significant differences were found between IES-R subscale scores (η^2^ = 0.047, *P* = .009). Participants experienced more distress from intrusions than from avoidance (0.269; 95% CI, 0.077-0.462; *P* = .006) or hyperarousal (0.233; 95% CI, 0.101-0.365; *P* < .001). Differences in distress resulting from hyperarousal and avoidance were not statistically significant (0.036; 95% CI, −0.164 to 0.237; *P* = .72). The follow-up (n = 96) and dropout group (n = 20) did not differ regarding IES-R raw sum scores (*U* = 696.00, *z* = −0.719, *P* = .47). A correlation matrix of the trauma-related variables at baseline and follow-up can be found in Table 2.

**Table 2.** Correlation Matrix of Trauma-Related Variables (Pearson Correlation Coefficients)

Variable	Baseline				Follow-up										
	IES-R raw sum score	Intrusion	Avoidance	Hyperarousal	IES-R raw sum score	Intrusion	Avoidance	Hyperarousal	Employment	Literacy in German	Time spent in captivity	Age	Change in faith	Change in social relationships	Change in IES-R subscale sum scores
Baseline															
IES-R raw sum score	1														
Intrusion	0.875^a^	1													
Avoidance	0.706^a^	0.359^a^	1												
Hyperarousal	0.808^a^	0.713^a^	0.289^a^	1											
Follow-up															
IES-R raw sum score	0.462^a^	0.507^a^	0.161	0.401^a^	1										
Intrusion	0.362^a^	0.489^a^	−0.072	0.439^a^	0.823^a^	1									
Avoidance	0.188	0.089	0.365^a^	−0.055	0.461^a^	−0.035	1								
Hyperarousal	0.409^a^	0.474^a^	0.042	0.450^a^	0.801^a^	0.738^a^	−0.026	1							
Employment	0.175	0.048	0.211^b^	0.164	0.040	−0.066	0.124	0.026	1						
Literacy in German	0.049	−0.015	0.063	0.083	−0.021	−0.105	0.064	−0.012	0.103	1					
Time spent in captivity	−0.073	0.002	−0.064	−0.134	0.186	0.171	0.184	0.069	−0.085	−0.115	1				
Age	−0.009	0.084	−0.098	−0.018	0.076	0.112	0.022	0.035	−0.123	−0.401^a^	0.310^a^	1			
Change in faith	0.143	0.093	0.170	0.071	−0.162	−0.087	−0.111	−0.125	0.102	−0.219^b^	−0.109	0.069	1		
Change in social relationships	−0.045	−0.062	0.042	−0.095	−0.242^b^	−0.302^a^	0.121	−0.339^a^	−0.064	0.071	0.034	0.038	0.062	1	
Change in IES-R subscale sum scores	−0.543^a^	−0.376^a^	−0.473^a^	−0.418^a^	0.491^a^	0.409^a^	0.228^b^	0.381^a^	−0.143	−0.121	0.260^b^	0.147	−0.268^b^	−0.209^b^	1

Abbreviation: IES-R, Impact of Event Scale–Revised.

^a^ *P* < .01.

^b^ *P* < .05.

#### Coping

At baseline, statistically significant differences were evident in perceived helpfulness of different coping strategies (η^2^ = 0.145, *P* < .001) (Table 3). Most participants reported a strengthening in faith resulting from trauma survival (strengthened, 59 [53.2%]; somewhat strengthened, 4 [3.6%]; no change, 38 [34.2%]; somewhat weakened, 5 [4.5%]; weakened, 5 [4.5%]) and felt no exclusion from the Yazidi community (not at all, 83 [78.3%]; a little bit, 0 [0%]; moderately, 10 [9.4%]; quite a bit, 2 [1.9%]; and extremely, 11 [10.4%]).

**Table 3.** Changes in Perceived Helpfulness of Posttraumatic Coping Strategies Over Time

Coping strategy^a^	Baseline, mean (SD)	Follow-up, mean (SD)	*P* value
Praying	3.29 (1.25)	3.01 (1.38)	.05
Believe in collective strength	3.01 (1.46)	2.90 (1.55)	.63
Believe in personal strength	2.71 (1.57)	3.22 (1.23)	.005
Seeking help within the Yazidi community	2.40 (1.68)	2.41 (1.76)	.96
Exchange trauma contents with others	2.18 (1.71)	1.99 (1.72)	.41
Seeking professional help	2.11 (1.72)	2.25 (1.76)	.45
Social retreat	1.64 (1.66)	1.74 (1.75)	.64

^a^ The helpfulness of 7 strategies in coping with symptoms of trauma was rated on a 5-point Likert scale (0-4), with 0 indicating not helpful at all and 4 indicating extremely helpful.

### Follow-up Assessment

#### Education and Employment

At follow-up, 51 women (53.1%) considered themselves literate in German, 47 (49.0%) in Arabic, and 30 (31.3%) in Kurdish-Kurmanji. Enrollment in school was reported by 60 participants (62.5%) (German language course, 44 [73.3%]; regular school, 16 [26.7%]). Table 1 indicates that the rates of literacy, employment, and employment seeking at follow-up were increased but school enrollment was decreased, likely because of participants graduating from a German language course. In Iraq, 13 (13.7%) of the participating women had worked outside their homes (at baseline, 15 women [12.9%] of the baseline sample).

#### Posttraumatic Stress Disorder

At follow-up, the mean (SD) IES-R raw sum score was 59.98 (14.55), with 88 (94.7%) scoring above a cutoff of 33. No statistically significant changes occurred compared with baseline IES-R raw sum scores (*t*_89_ = 0.270, *P* = .79) or subscale scores for avoidance (*t*_89_ = 0.158, *P* = .87), intrusions (*t*_90_ = −0.014, *P* = .99), or hyperarousal (*t*_90_ = 0.257, *P* = .80). By depicting the PTSD course during a 1-year period, mean (SD) changes were −0.44 (15.62) (range, −37.0 to 43.0) for IES-R raw sum scores (follow-up minus baseline) and −0.065 (2.14) (range, −5.21 to 5.83) for IES-R subscale sum scores (follow-up minus baseline). All IES-R scores at baseline and follow-up are presented in Table 1.

#### Associations With PTSD Severity at Follow-up

The data met the assumptions for regression analysis (Durbin-Watson statistic = 2.099). The analysis found that the model of symptom severity of intrusions, avoidance, and hyperarousal at baseline, age, and the number of days spent in captivity was statistically significantly associated with PTSD severity at follow-up (*R*^2^ = 0.312, adjusted *R*^2^ = 0.270, *P* < .001). As given in Table 4, standardized β values are highest for baseline intrusions (β = 0.389, *P* = .007) and number of days spent in captivity (β = 0.218, *P* = .02).

**Table 4.** Multiple Linear Regression Analysis With PTSD Severity at Follow-up (IES-R Subscale Sum Score) as the Dependent Variable

Variable	No. of participants	Mean *b* (SE) [95% CI]	β	*P* value	Tolerance
Constant	87	3.548 (1.204) [1.152 to 5.944]	NA	.004	NA
Baseline					
Avoidance	87	0.015 (0.234) [−0.451 to 0.481]	0.006	.95	0.882
Intrusions	87	0.829 (0.301) [0.230 to 1.427]	0.389	.007	0.426
Hyperarousal	87	0.366 (0.317) [−0.264 to 0.996]	0.159	.25	0.451
Age	87	0.010 (0.025) [−0.039 to 0.059]	0.038	.69	0.925
Time spent in captivity	87	0.004 (0.002) [0.001 to 0.007]	0.218	.02	0.925

Abbreviations: IES-R, Impact of Event Scale–Revised; NA, not applicable.

#### Coping

Over time, 2 statistically significant changes occurred in participants’ ratings of coping strategies (Table 3). Mean (SD) ratings of prayer (3.29 [1.25] at baseline and 3.01 [1.38] at follow-up, *P* = .05), belief in collective strength (3.01 [1.46] at baseline and 2.90 [1.55] at follow-up, *P* = .63), and belief in personal strength (2.71 [1.57] at baseline and 3.22 [1.23] at follow-up, *P* = .005) were rated most helpful, with no statistically significant differences in preference among these 3 (Table 4). Moreover, half of the participants reported a strengthening in social relationships during the past year (stronger relationships, 32 [34.0%]; somewhat stronger, 15 [16.0%]; no change, 31 [33.0%]; somewhat weakened; 7 [7.4%]; and weakened relationships, 9 [9.6%]).

#### Associations With PTSD Course

A multiple linear regression analysis was performed to assess the extent to which the number of days spent in captivity as well as posttraumatic changes in faith and social relationships could explain the variance of PTSD symptom course. To control for relevant sociodemographic influences, age was included in the model.^53^

The data met the assumptions for regression analysis (Durbin-Watson statistic = 2.190). A statistically significant regression equation was found (*R*^2^ = 0.177, adjusted *R*^2^ = 0.136, *P* = .003). Results are given in Table 5 and show an association of strengthening in faith (β = −0.206, *P* = .05) and social relationships (β = −0.221, *P* = .03) with PTSD symptom relief and an association between more days spent in captivity and PTSD aggravation (β = 0.227, *P* = .04).

**Table 5.** Multiple Linear Regression Analysis With PTSD Course (Change in IES-R Subscale Sum Score^a^) as the Dependent Variable

Variable	No. of participants	Mean *b* (SE) [95% CI]	β	*P* value	Tolerance
Constant	86			.91	
Change in faith	86	−0.353 (0.177) [−0.706 to −0.001]	−0.206	.05	0.949
Change in social relationships	86	−0.368 (0.169) [−0.704 to −0.032]	−0.221	.03	0.989
Time spent in captivity	86	0.004 (0.002) [0.0003 to 0.008]	0.227	.04	0.902
Age	86	0.028 (0.028) [−0.027 to 0.084]	0.105	.32	0.947

Abbreviations: IES-R, Impact of Event Scale–Revised; PTSD, posttraumatic stress disorder.

^a^ Positive change in IES-R subscale sum score indicates PTSD aggravation over time; negative change in IES-R subscale sum score indicates symptom decrease over time.

## Discussion

This cohort study investigated PTSD severity and coping in female genocide survivors 2 and 3 years after resettlement. Findings suggest that survivors experience severe psychological symptoms for years, even when participating in a HAP that aims to reduce postmigration stressors and provides mental health care. Longer captivity and severe intrusions are associated with unfavorable PTSD course, whereas a strengthening in faith through the traumatic event and positive changes in social relationships were identified as protective factors.

Even though approximately 73% of participants accepted psychotherapeutic help within the first 2 years of the program,^39^ most continued to experience high distress from PTSD symptoms up to 3 years after resettlement. This chronicity is consistent with research investigating different samples of refugees^14,56^ and genocide survivors.^8,9,10^ Moreover, the severity of PTSD in the current sample is comparable to Rwandan genocide survivors who experienced traumatic crisis during commemoration activities 16 years after the genocide (IES-R raw sum scores, 59-62).^57^

Distress resulting from intrusions, hyperarousal, and avoidance behavior each remained persistently high over time. However, the findings of this study suggest a special focus on intrusions when identifying high-risk groups in refugees because intrusions were rated as the most distressing of the PTSD symptom clusters. Previous research with other samples found acute intrusions to be associated with other PTSD symptoms shortly after a traumatic event^58^ and associated with PTSD 6 months later.^24^ The current study suggests that this association can still be found in intrusions of highly traumatized women years after the traumatic event. Avoidance and hyperarousal were not associated with PTSD severity 1 year later, although previous studies^20,21,22,23^ with other samples found such associations. This heterogeneity in results might be explained by different types of traumatic events and PTSD severity. As McFarlane^59^ argued, different courses of PTSD can be expected, depending on the initial severity of the traumatic event.

As another main finding, the amount of time spent in captivity is associated with PTSD severity 3 years after resettlement and with symptom intensification in the resettlement environment. This finding supports the theory of the building-block effect,^60^ meaning a dose effect between the number of experienced traumatic events and PTSD severity. With a mean of 6.8 months in captivity and severe PTSD symptoms 3 years after resettlement to a safe environment, the study’s findings suggest that this dose-response association exists long term even on the upper end of the PTSD severity spectrum and applies, besides the number of traumatic events,^42^ also for the amount of time spent in a traumatic situation.

The coping strategies perceived as most helpful in the current sample underline the importance of religion, community, and self-efficacy in the aftermath of trauma, which supports earlier qualitative findings.^31,32^ With only 2 significant changes over time, these preferences in coping can be interpreted as robust findings. Interestingly, the helpfulness ratings of prayer decreased, whereas self-efficacy, or belief in personal strength, increased during 1 year. Perceived personal strengthening might be a sign of adaptation to the new life in Germany and aligns with the current study’s findings that more women were literate, employed, and seeking employment than at the initial assessment. Even though the perceived helpfulness of prayer decreased slightly within the study’s assessment period, praying was still considered one of the most helpful ways to cope with trauma 3 years after resettlement. Moreover, most women reported an increase in faith through the experienced trauma, which could be identified as a protective factor regarding PTSD chronicity.

Drawing strength from the community was found to be another highly preferred coping strategy, whereas social retreat was considered least helpful at both assessment times. Moreover, an improvement in social relationships was associated with PTSD symptom relief. This result supports a previous systematic review^61^ that found that social support is well established as an important factor for trauma recovery. Because survivors of war-time rape are often rejected by their community and family,^2,62^ the current finding can be seen as an encouragement to focus on community-based interventions^63^ that strengthen social relationships in traumatized refugees.

### Limitations

This study has several limitations. Because of the preselected sample of HAP beneficiaries as particularly affected by severe and enduring trauma by IS fighters and the voluntary recruitment method, generalization to other populations might be limited.^34^ Moreover, because the IES-R is not a diagnostic tool, a valid PTSD prevalence rate could not be assessed in the current study. Future studies could benefit from a clinically assessed PTSD diagnosis. Limitations based on the use of translated versions of questionnaires and self-developed questionnaire items in an interpreter-aided interview setting should be considered when interpreting these results. However, because participants’ answers were given verbally in Kurdish and were translated afterward, errors in data collection attributable to interpreters’ spontaneous translation in the interview should be limited. Nevertheless, a bias in answers attributable to social desirability in the interview setting cannot be fully ruled out.

## Conclusions

The current study is unique because it depicts the longitudinal course of PTSD in a homogeneous sample in a setting in which postmigration stressors are limited and mental health services are available. Findings suggest that female refugee survivors of captivity are at high risk for severe and chronic PTSD beyond the initial resettlement period. The results also suggest that specifically assessing and targeting symptoms of intrusion while simultaneously fostering self-efficacy, faith, and social support may be promising strategies for similar samples in psychotherapy, as community-based interventions, and/or as scalable interventions for a global use. These strategies should be further investigated in future longitudinal studies.

## Notes

Supplement. **eAppendix.** Questionnaire Items Developed for the Study
