# Supplementary material for: Longitudinal Changes in Posttraumatic Stress Disorder After Resettlement Among Yazidi Female Refugees Exposed to Violence
Source: JAMA Netw Open. 2021 May 28;4(5):e2111120. doi: 10.1001/jamanetworkopen.2021.11120 (PMC8164098; doi:10.1001/jamanetworkopen.2021.11120)
Supplement: Supplement. — eAppendix. Questionnaire Items Developed for the Study [file jamanetwopen-e2111120-s001.pdf]

Supplementary Online Content

Denkinger JK, Rometsch C, Engelhardt M, et al. Longitudinal changes in posttraumatic stress disorder after resettlement among Yazidi female refugees exposed to violence. *JAMA Netw Open*. 2021;4(5):e2111120. doi:10.1001/jamanetworkopen.2021.11120

eAppendix. Questionnaire Items Developed for the Study

This supplementary material has been provided by the authors to give readers additional information about their work.

© 2021 Denkinger JK et al. *JAMA Network Open.*

eAppendix. Questionnaire Items Developed for the Study

Sociodemographic characteristics

How old are you?
What is your marital status?
If married or in a partnership, where is your spouse or partner?

Education

Can you read and write a simple message in any language?
What is your highest education level?
Please specify your highest education level
Are you currently in school?
If not, do you plan to attend school in the future?
Are you currently employed?
If yes, what is your current work?
If not, are you seeking employment?
If not, why not?
And before ISIS, were you employed outside of your home?
If yes, what was your work?

Religion and Ethnicity

What is your religious faith, if any?
How would you identify your ethnic group?

Trauma and effects of trauma

How long did you spend in captivity?	
How much do you feel excluded from the Yazidi community?	0 Not at all<br>1 A little bit<br>2 Moderately<br>3 Quite a bit<br>4 Extremely
How did your experience of violence by ISIS affect your faith?	0 Weakened<br>1 Somewhat weakened<br>2 No change<br>3 Somewhat strengthened<br>4 Strengthened

Coping

How much do the following strategies help you cope with the effects of the ISIS violence?	
Believe in collective strength (e.g. strength of the Yazidi community or your family)	0 Not at all<br>1 A little bit<br>2 Moderately<br>3 Quite a bit<br>4 Extremely
Believe in personal strength (e.g. believe in yourself or your own strength)	0 Not at all<br>1 A little bit<br>2 Moderately<br>3 Quite a bit<br>4 Extremely
Praying	0 Not at all<br>1 A little bit<br>2 Moderately<br>3 Quite a bit<br>4 Extremely
Retreat (e.g. spending time alone)	0 Not at all<br>1 A little bit<br>2 Moderately<br>3 Quite a bit<br>4 Extremely
Exchange trauma contents with others	0 Not at all<br>1 A little bit<br>2 Moderately<br>3 Quite a bit<br>4 Extremely
Seeking professional help (e.g. doctors, psycho-therapists)	0 Not at all<br>1 A little bit<br>2 Moderately<br>3 Quite a bit<br>4 Extremely
Seeking help within the Yazidi community	0 Not at all<br>1 A little bit<br>2 Moderately<br>3 Quite a bit<br>4 Extremely
Do you have any other strategies that help you cope?	

© 2021 Denkinger JK et al. *JAMA Network Open.*

Additional questions in T2

added based on the experiences made in T1

Are you attending language classes only or are you enrolled in regular school, or both?	
If literate, in which language can you read and/or write?	
Have you noticed any change in social and family relationships, including with your children, over the last year? Did your relationships become better or worse?	0 Worse<br>1 Somewhat worse<br>2 Did not change<br>3 Somewhat better<br>4 Better

© 2021 Denkinger JK et al. *JAMA Network Open.*
